# Synthesis of ZnFe_2_O_4_ Nanospheres with Tunable Morphology for Lithium Storage †

**DOI:** 10.3390/nano13243126

**Published:** 2023-12-13

**Authors:** Filipp S. Volkov, Mikhail A. Kamenskii, Elena G. Tolstopjatova, Lusine A. Voskanyan, Natalia P. Bobrysheva, Olga M. Osmolovskaya, Svetlana N. Eliseeva

**Affiliations:** Institute of Chemistry, Saint Petersburg State University, 7/9 Universitetskaya Nab., 199034 Saint Petersburg, Russia; sokkorat@gmail.com (F.S.V.); kamenskymisha@yandex.ru (M.A.K.); e.tolstopyatova@spbu.ru (E.G.T.); o.osmolovskaya@spbu.ru (O.M.O.)

**Keywords:** lithium-ion batteries, anode materials, zinc ferrite ZnFe_2_O_4_, electrochemical performance

## Abstract

ZnFe_2_O_4_ (ZFO) nanospheres with complex structures have been synthesized by a one-step simple solvothermal method using two different types of precursors—metal chlorides and nitrates —and were fully characterized by XRD, SEM, XPS, and EDS. The ZFO nanospheres synthesized from chloride salts (ZFO_C) were loose with a size range of 100–200 nm, while the ZFO nanospheres synthesized from nitrate salts (ZFO_N) were dense with a size range of 300–500 nm but consisted of smaller nanoplates. The different morphologies may be caused by the different hydrolysis rates and different stabilizing effects of chloride and nitrate ions interacting with the facets of forming nanoparticles. Electrochemical tests of nitrate-based ZFO nanospheres as anode materials for lithium-ion batteries demonstrated their higher cyclic stability. The ZFO_C and ZFO_N samples have initial specific discharge/charge capacities of 1354/1020 and 1357/954 mAh∙g^−1^, respectively, with coulombic efficiencies of 75% and 71%. By the 100th cycle, ZFO_N has a capacity of 276 mAh∙g^−1^, and for ZFO_C, only 210 mAh∙g^−1^ remains after 100 cycles.

## 1. Introduction

Metal ferrites MFe_2_O_4_ (M = Co, Mn, Zn, etc.) are attracting researchers’ attention due to different application fields like photocatalysis, water splitting, semiconductor materials, electrode materials for metal-ion batteries and other [1,2,3]. Among them, zinc ferrite ZnFe_2_O_4_ (ZFO) stands out due to its environmental friendliness, high structural and thermal stability. Furthermore, different synthetic routes allow to obtain ZFO with different crystallinity and morphology, and thus to control its properties.

In the field of energy storage and lithium-ion batteries (LIBs), ZnFe_2_O_4_-based anode materials have an extremely high theoretical capacity value of more than 1000 mAh∙g^−1^ due to the conversion reaction mechanism with total nine-electron transfer. On the other hand, the drawbacks of ZFO-based anode materials associated with the conversion reaction are first-cycle irreversibility, cyclic instability, and the high volume changes during the lithiation/delithiation process [4,5,6]. In addition, ZFO-based anode materials suffer from low electric conductivity. 

The mechanism of the lithiation/delithiation process and the overall electrochemical performance of the material are dependent on the morphology of the initial ZFO [7]. As a rule, materials with smaller average crystallite sizes can reduce the Li-ion diffusion length, allowing faster charge/discharge rates. 

To improve the properties of the ZFO electrode materials and minimize their drawbacks, two main strategies are applied: preparation of nanomaterials with given morphology and development of composite materials. Reducing the particle size leads to an increase in the real surface area, improves the contact between the electrode and the electrolyte, and shortens the diffusion paths of lithium ions and electrons. Various types of one-dimensional [5], two-dimensional [8], and three-dimensional ZFO materials [9,10,11] have been prepared and investigated as anode materials for Li-ion batteries. Another interesting solution is the preparation of hollow materials with high internal spaces and an appropriate surface area, which facilitates diffusion processes [12].

In this work, we used a simple one-step solvothermal route to obtain ZFO nanospheres from nitrate salts, which allowed us to take advantage of the lower hydrophilicity of iron and zinc nitrates. Although there are several works in which nitrate salts have been used as precursors for the synthesis of ZFO, it is difficult to compare data on the electrochemical performance of materials obtained under different synthesis conditions. In our study, the use of nitrates allowed to obtain ZFO anode materials with slightly improved cycling stability compared to those obtained under the same conditions of solvothermal synthesis using chloride salts as precursors.

## 2. Materials and Methods

### 2.1. Synthesis of ZnFe_2_O_4_

Zinc ferrite was prepared by a simple one-step solvothermal reaction [12]. 4 mmol FeCl_3_∙6H_2_O and 2 mmol ZnCl_2_ were dissolved in ethylene glycol under continuous stirring. Then, 4.8 g of sodium acetate trihydrate (CH_3_COONa∙3H_2_O) was slowly added to the solution and continued stirring for one hour. The resulting solution was transferred to 40 mL Teflon-lined stainless-steel autoclave and heated in an oven at 200 °C for 15 h. After cooling to room temperature, the resulting product was separated by centrifugation (4700 rpm for 5 min), washed several times with ethanol and water and dried in a vacuum oven at 60 °C for 12 h. The brownish powder obtained was designated as ZFO_C. For the synthesis of ZFO_N, a similar procedure was used, except that zinc and iron nitrates (ZnNO_3_)_2_∙6H_2_O and Fe(NO_3_)_3_∙9H_2_O were used instead of zinc and iron chlorides.

### 2.2. Materials Characterization

X-ray diffraction (XRD) patterns were recorded using a Cu-Kα radiation on a D2 Phaser diffractometer (Bruker, Berlin, Germany) in the range of 10–80°. The lattice parameters were determined using the Pauli method in Topas 4.2 software (Bruker AXS, Germany), and the average crystallite sizes were calculated from the XRD data according to the Scherrer equation.

The morphology of the as-prepared ZFO samples was studied by scanning electron microscopy (SEM) using a SUPRA 40VP (Carl Zeiss, Jena, Germany) microscope and by transmission electron microscopy (TEM) using Zeiss Libra 200FE (Carl Zeiss, Germany) microscope, equipped with an X-Max Analytical Silicon Drift Detector for EDS analysis.

The X-ray photoelectron spectra (XPS) were measured on the Escalab 250Xi spectrometer (Thermo Fisher Scientific Inc., Waltham, MA, USA) equipped with an Al Kα source (photon energy of 1486.6 eV).

### 2.3. Electrochemical Tests

The electrode materials were prepared by mechanically mixing electroactive material (ZFO_C or ZFO_N), carbon black and PVDF dissolved in NMP in the following ratio: 70 wt.% of ZFO, 20 wt.% of C and 10 wt.% of PVDF. The resulting viscous slurry was cast onto the copper foil with a 150 μm gap applicator and dried under vacuum at 80 °C for 12 h. The resulting electrode material was roll-pressed and cut into disks of ⌀ 12 mm. The mass loading of the electroactive material was in the range of ≈1 mg∙cm^−2^. The ZFO anodes were assembled in two-electrode CR2032 coin cells vs. Li foil (Alfa Aesar, Haverhill, MA, USA), using Celgard^®^2325 polypropylene membrane as separator and commercial electrolyte 1 M LiPF_6_ in EC:DEC:DMC (ethylene carbonate:diethyl carbonate:dimethyl carbonate 1:1:1 vol.%). 

The electrochemical properties of the electrode materials were evaluated by galvanostatic charge–discharge (GCD) and cyclic voltammetry (CV) in the potential range of 0.01–3.0 V vs. Li/Li^+^. GCD curves were obtained on an automatic battery test system CT-4008 (Neware Co., Shenzhen, China) at a current density of 0.2 A∙g^−1^. CV measurements were performed on a BCS-805 potentiostat/galvanostat (Biologic, Seyssinet-Pariset, France) at a scan rate of 0.2 mV∙s^−1^.

## 3. Results and Discussion

### 3.1. Physical Characterization

**Figure 1:**
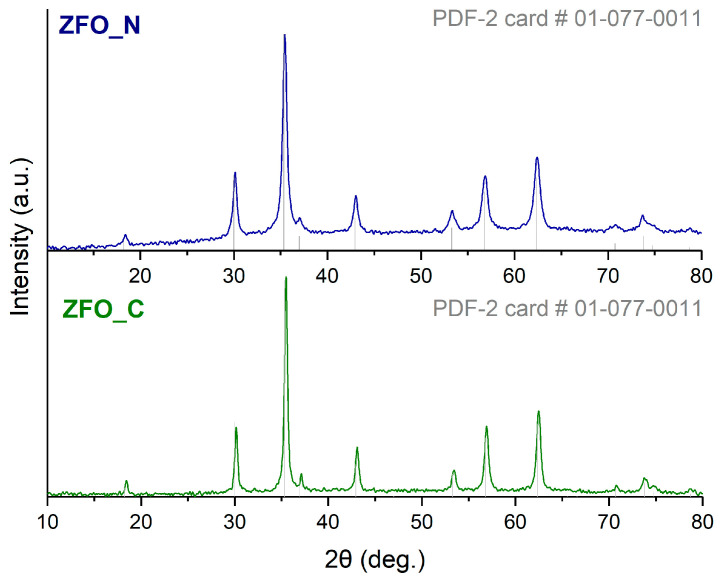
XRD patterns of zinc ferrite samples (ICDD ZnFe_2_O_4_ card #00-077-0011) synthesized in presence of chloride (green curve) and nitrate (blue curve) anions.

The as-prepared ZFO samples were first characterized by X-ray diffraction (Figure 1). Both synthetic routes allow one to obtain only the ZnFe_2_O_4_ phase without any impurities. The XRD pattern showed that the ZFO_N sample was less crystallized in comparison with the ZFO_C sample. The lattice parameters and crystallite sizes, calculated from the Scherrer equation, are given in Table 1. The ZFO_C nanospheres were less dense and had a higher value of crystallite size.

**Figure 2:**
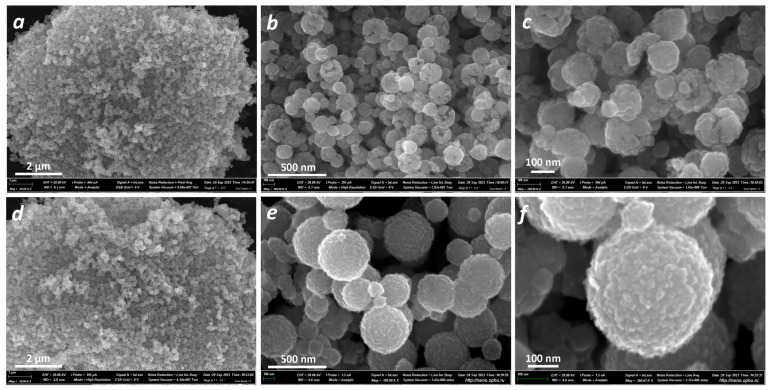
SEM images of ZnFe_2_O_4_ samples at different magnifications: ZFO_C (**a**–**c**) and ZFO_N (**d**–**f**).

The morphology of the samples was further investigated by scanning electron microscopy (Figure 2). For the ZFO_C sample, small nanospheres with an average size of 100–200 nm were observed (Figure 2a). It appears that these spheres are porous (Figure 2b) and consist of aggregated nanoplates (Figure 2c). The ZFO_N sample also consists of nanospheres, but with a diameter of approximately 300–500 nm (Figure 2d). These spheres are dense (Figure 2e) and are also composed of nanoplates (Figure 2f) with sizes smaller than those of the ZFO_C sample. A smoother and more rounded shape is observed for ZFO_N compared to the ZFO_C samples.

**Figure 3:**
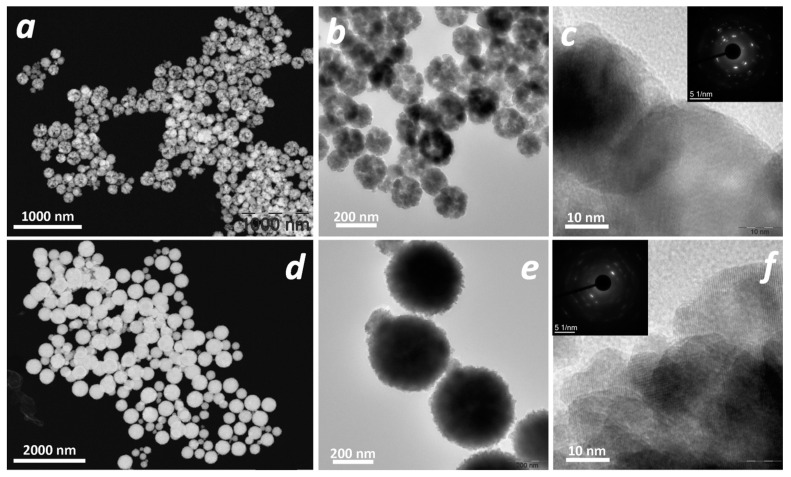
HAADF images (**a**,**d**), TEM images (**b**,**e**) and HR-TEM images ((**c**,**f**)—SAED on the insert) for ZFO_C (**a**–**c**) and ZFO_N (**d**–**f**).

To carefully study the structure of the as-prepared samples, an additional TEM investigation was performed (Figure 3). The HAADF-STEM data for the ZFO samples (Figure 3a,d) provide a clearer visualization of the surface features of the nanoplates consisting of nanospheres, confirming our assumption that the spheres are loose for the ZFO_C sample and dense for the ZFO_N sample. Indeed, this is clearly visible in Figure 3a,d. This can also be seen in Figure 3b,e at a higher magnification. The nanoplate sizes were larger in the case of ZFO_C (Figure 3c,f), and both samples were polycrystalline (the rings are clearly visible in the SAED images, Figure S1). In addition, the crystal planes identified in the HRTEM images (Figure S1a,c) are in good agreement with the SAED data (Figure S1b,d).

**Figure 4:**
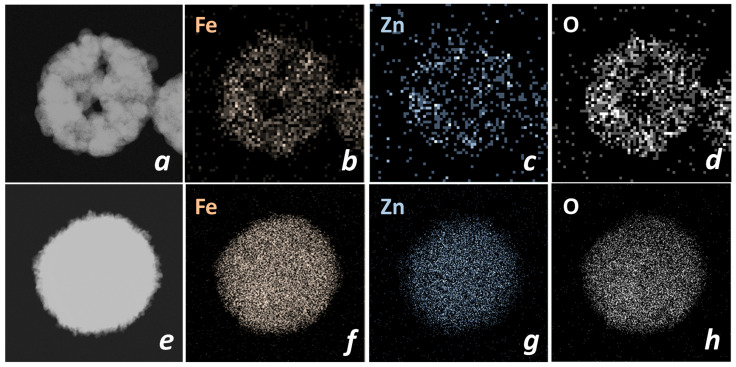
EDS maps of ZFO_C (**a**–**d**) and ZFO_N (**e**–**h**) samples: TEM images (**a**,**e**), Fe map (**b**,**f**), Zn map (**c**,**g**), O map (**d**,**h**).

The distribution of Fe, Zn, and O atoms in the volume of the obtained nanospheres is shown in Figure 4. It can be seen that for the ZFO_N sample, all three maps show a uniform distribution of atoms, whereas for the ZFO_C sample, it is quite pronounced only for Fe and O. The most likely reason for this phenomenon could be non-stoichiometry, i.e., a higher iron content. Indeed, the EDX results (Table 1, Figures S2 and S3) show that the Fe:Zn ratio is close to stoichiometric for the ZFO_N sample and exceeds the value of 2 units for the ZFO_C sample. The sample characteristics discussed above are summarized in Table 2.

The formation of spheres in both cases is related to the presence of ethylene glycol molecules in the reaction medium, which form oligomers under hydrothermal conditions and have a high affinity for the inorganic nanoparticle surface. They act as stabilizers and, in addition, as structuring agents that force the nanoplates to aggregate into a minimum energy form—the spherical. The difference in the agglomeration processes of nanoplates is apparently caused by two reasons—the different hydrolysis rates for chlorides and nitrates (in both cases, water is present in the reaction medium), as well as the screening effect of ions, which complicates the assembling of the plates along one of the facets and thus facilitates the process along other facets. Harsh conditions lead to the fixation of the formed structures and their further crystallization. Thus, by using precursors of different natures, it is possible to significantly influence the morphology of solvothermal synthesis products.

The XPS spectra of the ZFO_C and ZFO_N samples are shown in Figure S4. Characteristic peaks of Zn, Fe, O and C were detected in the survey spectra (Figure S4a,e).

### 3.2. Electrochemical Performance

ZnFe_2_O_4_ demonstrates a lithium insertion mechanism that involves both conversion and alloying reactions. After the conversion reaction of ZFO with lithium ions and the formation of metallic Zn, Fe, and Li_2_O, the resulting Zn can further react with lithium to form a LiZn alloy, thus contributing additional capacity.

The Li storage mechanism of ZFO is proposed as follows [4,5,6]: during the initial discharge

ZnFe_2_O_4_ + xLi^+^ + xe^−^ → Li_x_ZnFe_2_O_4_ [0.2 < x < 2] (1)

Li_x_ZnFe_2_O_4_ + (8 − x)Li^+^ + (8 − x)e^−^ ↔ Zn^0^ + 2Fe^0^ + 4Li_2_O (2)

Zn^0^ + Li^+^ + e^−^ ↔ LiZn (3)

and in the following recharge process, the reaction proceeds as follows:

Zn^0^ + Li_2_O ↔ ZnO + 2Li^+^ + 2e^−^ (4)

2Fe^0^ + 2Li_2_O ↔ Fe_2_O_3_ + 4Li^+^ + 4e^−^ (5)

**Figure 5:**
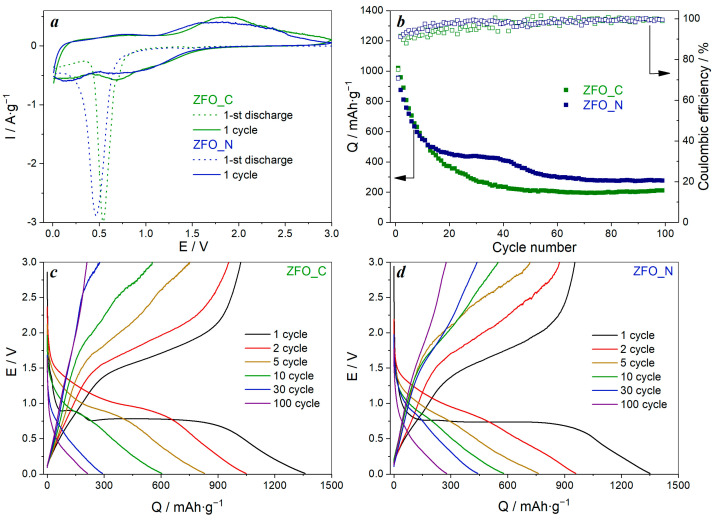
Cyclic voltammograms at a scan rate of 0.2 mV∙s^−1^ (**a**), cycle stability (**b**), charge/discharge profiles (**c**,**d**) at a current density of 0.2 A∙g^−1^ of ZnFe_2_O_4_-based anode materials.

The electrochemical performance of the ZnFe_2_O_4_ samples was investigated by cyclic voltammetry and galvanostatic charge/discharge (Figure 5). A sharp cathodic peak at a voltage of about 0.5–0.6 V is observed at the first discharge cycle (Figure 5a). This peak corresponds to the lithiation of ZFO with formation of Li_x_ZnFe_2_O_4_ (Equation (1)) and the reversible reduction of Zn^2+^ to Zn^0^ and Fe^3+^ to Fe^0^ during further lithiation of Li_x_ZnFe_2_O_4_ with the formation of Li_2_O, followed by the formation of the LiZn alloy (Equations (2) and (3)) and the solid electrolyte interphase (SEI) layer on the electrode surface due to the electrolyte decomposition [5,13]. For the first charge cycle (delithiation), a broad anodic peak at E ≈ 1.85 V is observed, corresponding to the disappearance of the LiZn alloy and the destruction of Li_2_O with the oxidation of Zn^0^ and Fe^0^ to ZnO and Fe_2_O_3_, respectively (Equations (4) and (5)). The cathodic peak at E ≈ 0.70 V, observed during the second discharge cycle corresponds to the reversible reduction reaction of amorphous ZnO and Fe_2_O_3_ [7].

The GCD tests of ZFO-based anode materials showed that the initial charge capacity values were 1020 mAh∙g^−1^ for ZFO_C, slightly higher than 955 mAh∙g^−1^ for ZFO_N (Figure 5b–d). The charge/discharge profiles of the ZFO anodes are presented in Figure 5c,d. During the first discharge, the main plateau at E = 0.75 V is observed for both anode materials, and in the case of ZFO_C, an additional plateau at E = 0.89 V is seen, which is absent for ZFO_N. Such behaviour can be attributed to Li^+^ intercalation into the ZnFe_2_O_4_ structure [14,15,16,17]. During the following cycles, the shape of the curves changes drastically to a quasi-linear form, indicating not only an electrochemical reaction with Li^+^ insertion, but also a pseudocapacitive response.

For both materials, a sharp capacity drop to about 500 mAh∙g^−1^ is observed during the first fifteen cycles (Figure 5b) due to conversion mechanism reaction of ZFO resulting in phase transformations [18]. During the following cycles, ZFO_N has the better cyclability, with an insignificant capacity fading after the 40th cycle and, as a result, 276 mAh∙g^−1^ is still observed at the 100th cycle. For ZFO_C, only 210 mAh∙g^−1^ remains after 100 cycles, and a slight capacity growth starts after the 70th cycle due to the continuous formation of the solid electrolyte interphase, which is electroactive due to non-faradaic reactions on its surface [19,20]. The coulombic efficiency of the first charge/discharge process is low, 75% and 71% for ZFO_C and ZFO_N, respectively, as usual for ZFO-based materials, but it increases in subsequent cycles to 100%. 

In order to evaluate the morphology of the ZFO_C and ZFO_N materials after the electrochemical tests, ex situ SEM images were taken (Figure S5). The SEM images show that both materials retain their morphology after cycling, although they become denser.

## 4. Conclusions

ZnFe_2_O_4_ nanospheres with complex structures were obtained via a simple solvothermal synthetic procedure from two types of precursors (zinc and iron chlorides and nitrates). The synthesis from the nitrate salts was easier to perform due to the lower hydrophilicity of the nitrates. It is shown that the regulation of nanosphere morphology parameters can be easily achieved by using different precursors. 

The nitrate-based ZFO nanospheres were larger than those synthesized from chlorides but consisted of smaller nanoplates. The formation of loose, small nanospheres in the case of chloride salts and dense, large nanospheres in the case of nitrate salts is assumed to be caused by the different hydrolysis rates and different stabilizing effects of chloride and nitrate ions interacting with the facets of forming nanoparticles. 

The electrochemical properties of both as-obtained ZFO materials tested under the same conditions were similar, but more stable electrochemical performance and higher capacity retention were observed for the nitrate-based ZFO electrodes. This, together with the convenience of the synthesis of nitrate salts, makes this synthesis route more promising for further applications. 

Since the electrochemical stability of the nitrate-based ZFO cathodes is slightly better, the next step is to prepare annealed and carbon-coated materials to achieve better long-term cycling stability of the material.

## Figures and Tables

**Table 1:** Summary of the XRD data for ZnFe_2_O_4_ samples: lattice parameter, crystallite size, Fe:Zn ratio according to EDX.

Sample	a (Å)	d_XRD_ (nm)	Fe Atomic%	Zn Atomic%	Fe:Zn Ratio
ZFO_C	8.426 (2)	19.8 (6)	24.84	9.93	2.37
ZFO_N	8.433 (2)	12.1 (4)	17.35	8.15	2.01

**Table 2:** The ZFO-sample characteristics.

Sample	Precursor	Shape	Size, nm	Structure	Stoichiometry	Nanoplates
ZFO_C	Chloride	Sphere	100–200	Loose	No	Large
ZFO_N	Nitrate	Sphere	300–500	Dense	Yes	Small

## Data Availability

The data presented in this study are available on request from the corresponding author.

## Supplementary Materials

The following supporting information can be downloaded at: https://www.mdpi.com/article/10.3390/nano13243126/s1, Figure S1: SAED data for ZFO_C (a) and ZFO_N (c) samples; HRTEM images for ZFO_C (b) and ZFO_N (d) samples; Figure S2: EDX spectrum of ZFO_C sample; Figure S3: EDX spectrum of ZFO_N sample; Figure S4: XPS spectra of ZFO_C (a–d) and ZFO_N (e–h) samples; Figure S5: SEM images of zinc ferrite-based electrodes before electrochemical tests: ZFO_C (a) and ZFO_N (c) and after cycling: ZFO_C (b) and ZFO_N (d). The following references are also mentioned in Supplementary Materials: [21,22,23,24,25].
