# Supplementary material for: Synthesis of ZnFe2O4 Nanospheres with Tunable Morphology for Lithium Storage
Source: Nanomaterials (Basel). 2023 Dec 13;13(24):3126. doi: 10.3390/nano13243126 (PMC10745651; doi:10.3390/nano13243126)
Supplement: Supplementary file 1 [file nanomaterials-13-03126-s001.zip › nanomaterials-2771501-supplementary.pdf]

nanomaterials

Supplementary Materials

Synthesis of $ZnFe_2O_4$ Nanospheres with Tunable Morphology for Lithium Storage †

Filipp S. Volkov, Mikhail A. Kamenskii, Elena G. Tolstopjatova, Lusine A. Voskanyan, Natalia P. Bobrysheva, Olga M. Osmolovskaya and Svetlana N. Eliseeva *

Institute of Chemistry, Saint Petersburg State University, 7/9 Universitetskaya Nab., 199034 Saint Petersburg, Russia; sokkorat@gmail.com (F.S.V.); kamenskymisha@yandex.ru (M.A.K.); e.tolstopyatova@spbu.ru (E.G.T.); o.osmolovskaya@spbu.ru (O.M.O.)

* Correspondence: svetlana.eliseeva@spbu.ru

† Dedicated to the 300th Anniversary of St. Petersburg University.

Citation: Volkov, F.S.; Kamenskii, M.A.; Tolstopjatova, E.G.; Voskanyan, L.A.; Bobrysheva, N.P.; Osmolovskaya, O.M.; Eliseeva, S.N. Synthesis of $ZnFe_2O_4$ Nanospheres with Tunable Morphology for Lithium Storage. *Nanomaterials* **2023**, *13*, 3126. https://doi.org/10.3390/ 10.3390/nano13243126

Academic Editor: Zhan'ao Tan

Received: 28 November 2023
Revised: 7 December 2023
Accepted: 11 December 2023
Published: 13 December 2023

Copyright: © 2023 by the authors. Submitted for possible open access publication under the terms and conditions of the Creative Commons Attribution (CC BY) license (https://creativecommons.org/licenses/by/4.0/).

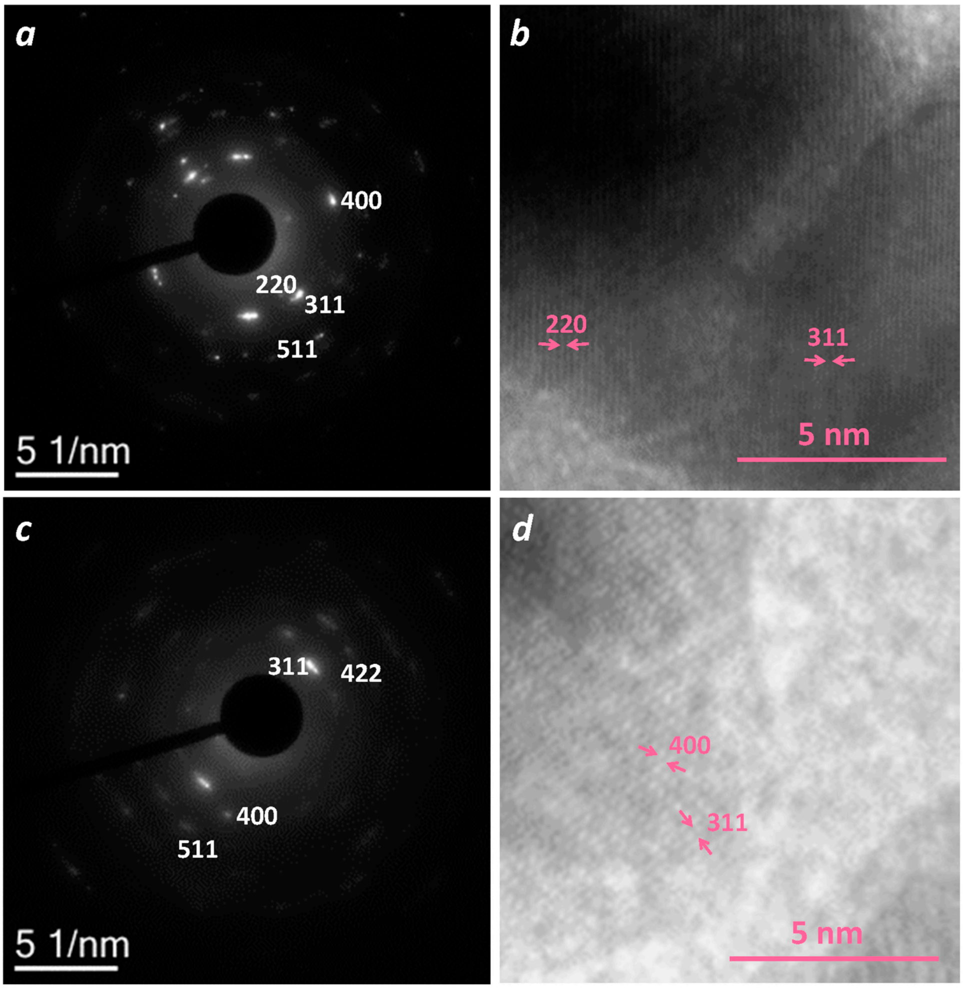

Figure S1. SAED data for ZFO_C (**a**) and ZFO_N (**c**) samples; HRTEM images for ZFO_C (**b**) and ZFO_N (**d**) samples.

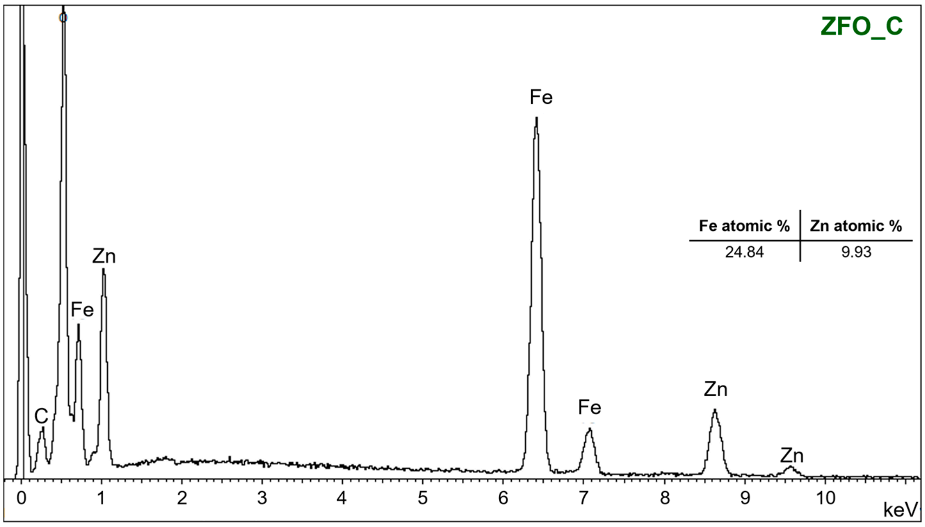

Figure S2. EDX spectrum of ZFO_C sample.

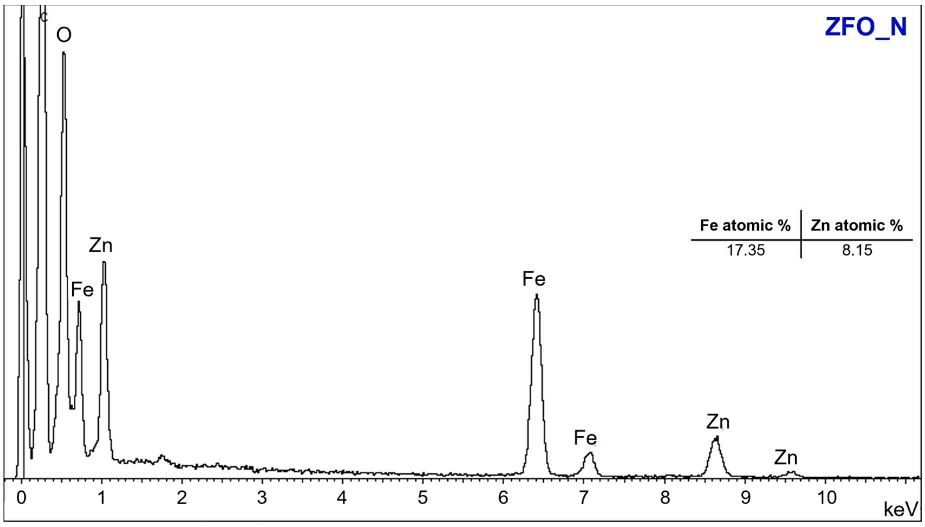

Figure S3. EDX spectrum of ZFO_N sample.

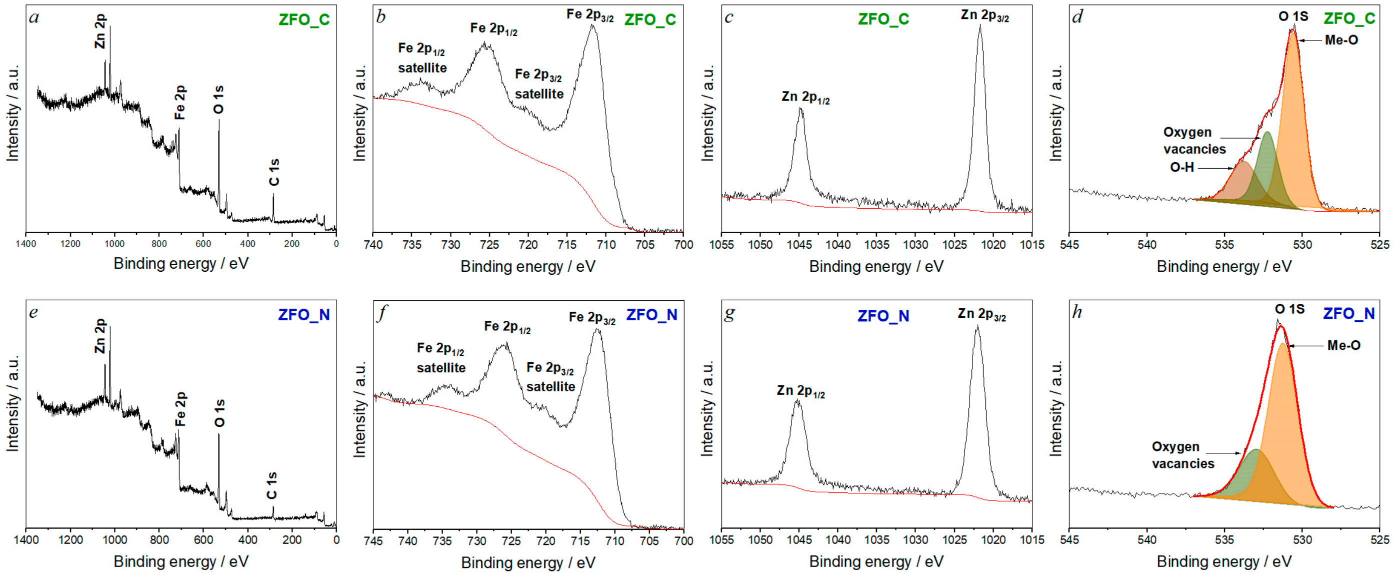

Figure S4. XPS spectra of ZFO_C (**a-d**) and ZFO_N (**e-h**) samples.

Figure S4 shows the XPS spectra of samples ZFO_C and ZFO_N. Characteristic peaks of Zn, Fe, O and C are detected in the survey spectra (Figure S4 a,e). For the Fe spectrum (Figure S4 b,f), four peaks were observed, two of which correspond to the Fe 2p1/2 and Fe 2p3/2 with a spin splitting of about 13.6 eV, as well as two satellite peaks corresponding to the +3 oxidation state of Fe [1]. The Zn 2p spectrum (Figure S4 c,g) shows two peaks with binding energies of 1044.8 and 1021.7 eV. The peak positions and spin splitting of 23 eV correspond to Zn in the +2 oxidation state [2,3]. In the case of the O 1s spectrum (Figure S4 d,h), mathematical processing allows the components of the oxygen peak to be detected. For the ZFO_N sample, two peaks are detected, one corresponding to oxygen in the zinc ferrite lattice and the other to oxygen vacancies [4,5]. For sample ZFO_N an additional peak corresponding to chemisorbed water or solvent molecules can be detected.

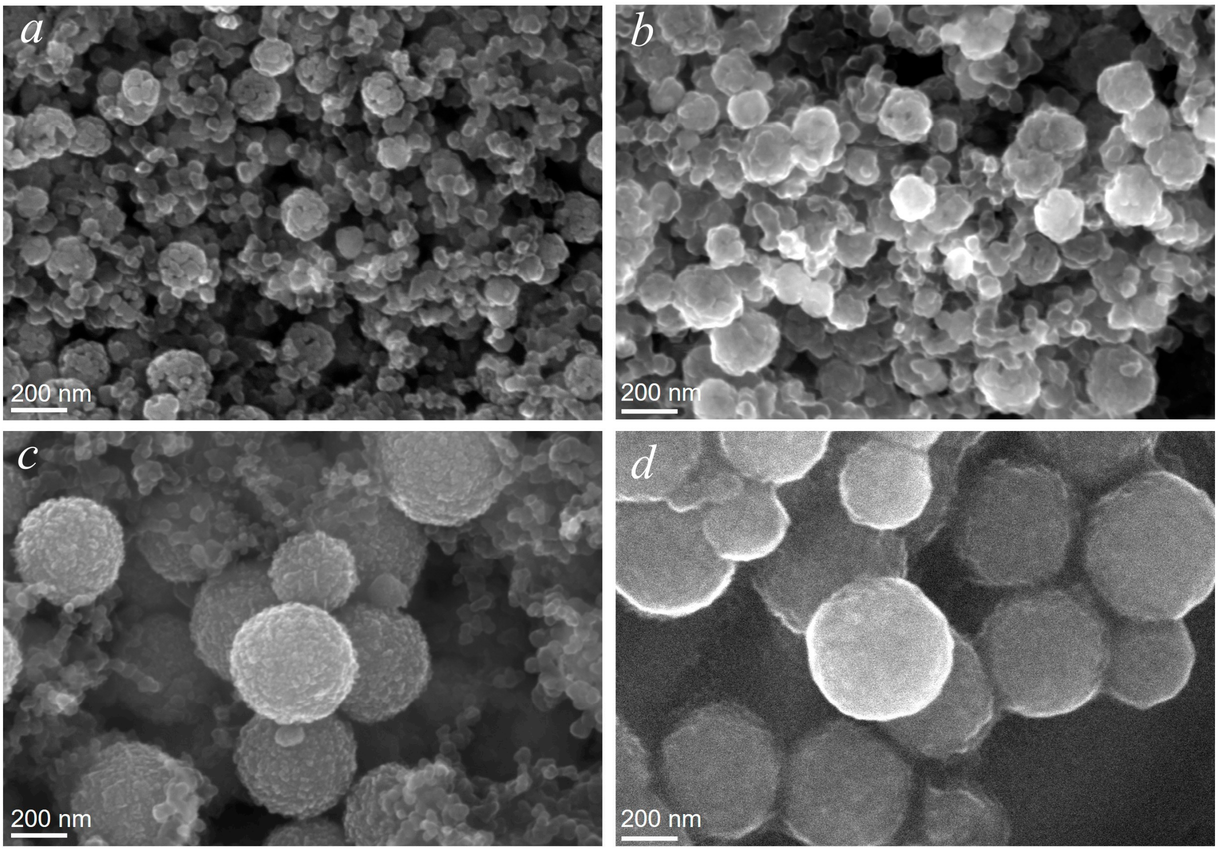

Figure S5. SEM images of zinc ferrite-based electrodes before electrochemical tests: ZFO_C (**a**) and ZFO_N (**c**) and after cycling: ZFO_C (**b**) and ZFO_N (**d**).

References

1. Poudel, M.B.; Kim, H.J. Confinement of Zn-Mg-Al-Layered Double Hydroxide and α -Fe_2O_3 Nanorods on Hollow Porous Carbon Nanofibers : A Free-Standing Electrode for Solid-State Symmetric Supercapacitors. *Chem. Eng. J.* **2022**, *429*, 132345, doi:10.1016/j.cej.2021.132345.
2. Rabin, N.N.; Morshed, J.; Akhter, H.; Islam, S.; Hossain, A.; Alam, M.; Karim, M.R.; Hasnat, M.A. Surface Modification of the ZnO Nanoparticles with γ -Aminopropyltriethoxysilane and Study of Their Photocatalytic Activity, Optical Properties and Antibacterial Activities. *Int. J. Chem. React. Eng.* **2016**, *14*, 785–794, doi:10.1515/ijcre-2015-0141.
3. Dai, L.; Strelow, C.; Kipp, T.; Mews, A.; Benkenstein, I.; Ei, D.; Vuong, T.H.; Rabeah, J.; Mcgettrick, J.; Lesyuk, R.; Klinke, C. Colloidal Manganese-Doped ZnS Nanoplatelets and Their Optical Properties. *Chem. Mater.* **2021**, *33*, 275–284. doi:10.1021/acs.chemmater.0c03755.
4. Kim, J.H.; Jang, Y.J.; Kim, J.H.; Jang, J.W.; Choi, S.H.; Lee, J.S. Defective $ZnFe_2O_4$ Nanorods with Oxygen Vacancy for Photoelectrochemical Water Splitting. *Nanoscale* **2015**, *7*, 19144–19151, doi:10.1039/c5nr05812k.
5. Al-Najar, B.; Younis, A.; Hazeem, L.; Sehar, S.; Rashdan, S.; Shaikh, M.N.; Albuflasa, H.; Hankins, N.P. Thermally Induced Oxygen Related Defects in Eco-Friendly $ZnFe_2O_4$ Nanoparticles for Enhanced Wastewater Treatment Efficiencies. *Chemosphere* **2022**, *288*, doi:10.1016/j.chemosphere.2021.132525.
